# Involvement of Mitochondrial Disorders in Septic Cardiomyopathy

**DOI:** 10.1155/2017/4076348

**Published:** 2017-10-22

**Authors:** Arthur Durand, Thibault Duburcq, Thibault Dekeyser, Remi Neviere, Michael Howsam, Raphael Favory, Sebastien Preau

**Affiliations:** ^1^Intensive Care Department, Université de Lille, Inserm, CHU Lille, U995, Lille Inflammation Research International Center (LIRIC), Lille, France; ^2^Département de Physiologie, CHU Martinique, Faculté de Médecine, Université des Antilles, 97200 Fort de France, France; ^3^Université de Lille, Inserm, U995, Lille Inflammation Research International Center (LIRIC), Lille, France

## Abstract

Sepsis is defined as a life-threatening organ dysfunction caused by a dysregulated host response to infection. It remains a leading cause of death worldwide, despite the development of various therapeutic strategies. Cardiac dysfunction, also referred to as septic cardiomyopathy, is a frequent and well-described complication of sepsis and associated with worse clinical outcomes. Recent research has increased our understanding of the role of mitochondrial dysfunction in the pathophysiology of septic cardiomyopathy. The purpose of this review is to present this evidence as a coherent whole and to highlight future research directions.

## 1. Introduction

Sepsis is defined by consensus as a life-threatening organ dysfunction caused by a dysregulated host response to infection [1]. For clinical operationalization, patients with sepsis can be identified by the presence of an infection and an increase in the sepsis-related organ failure assessment score of 2 points or more. With short-term mortality over 10%, septic patients require hospitalization in intermediate or intensive care units [2]. Septic shock is defined as a subset of sepsis in which particularly profound circulatory, cellular, and metabolic abnormalities are associated with a hospital mortality rate of over 40% [1]. Patients with septic shock can be clinically identified by a vasopressor requirement to maintain a mean arterial pressure of 65 mm Hg or greater and serum lactate level greater than 2 mmol/L in the absence of hypovolemia. Both sepsis and septic shock are frequent in Western countries, with an incidence ranging from 270 to 437 cases per 100000 inhabitants per year [3]. The mortality attributable to sepsis is 5.3 million people per year worldwide, representing around 10% of deaths [4].

**Figure 1:**
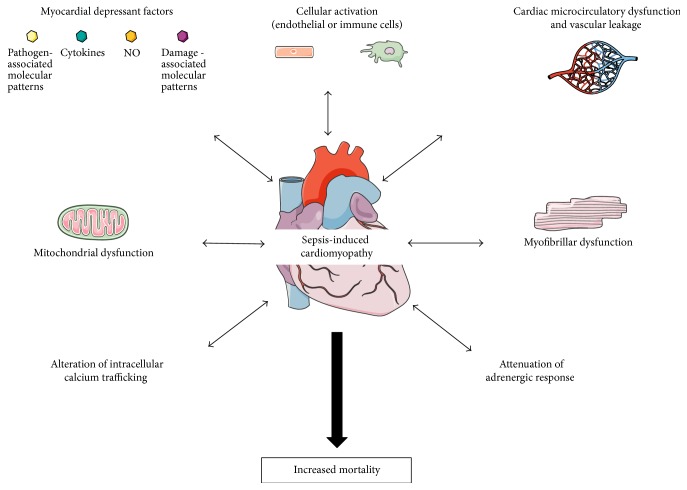
Main pathophysiological mechanisms of sepsis-induced cardiomyopathy. During sepsis, recognition of pathogen-associated molecular patterns by immune cells activates inflammation pathways and the release of myocardial depressant factors in the extracellular space. The subsequent activation of endothelial cells leads to alterations of microcirculatory perfusion and vascular leakage that are implicated in sepsis-induced myocardial dysfunction. Among intracellular mechanisms, myofibrillar dysfunction, alterations of calcium trafficking, attenuation of adrenergic response, and mitochondrial dysfunction seem to play important roles in sepsis-induced cardiomyocyte impairment. NO: nitric oxide.

The incidence of cardiac dysfunction in septic shock (septic cardiomyopathy) is between 40 and 60%, as diagnosed within the first 3 days [5]. The presence of cardiac dysfunction is associated with increased mortality in patients with sepsis: 28-day mortality of patients hospitalized for sepsis was 16% in absence and 47% in the presence of myocardial dysfunction [6]. Sepsis-related cardiomyopathy is clearly multifactorial (Figure 1()), and the general mechanisms of underlying this dysfunction have been reviewed elsewhere [7–10]. In summary, the potential extracellular candidates responsible for septic cardiomyopathy include pathogen-associated molecular patterns, cytokines, nitric oxide (NO), and damage-associated molecular patterns. Moreover, myocardial edema due to vascular leakage may also influence cardiac function in sepsis. At the cardiomyocyte level, mechanisms include the attenuation of the adrenergic response (i.e., downregulation of beta-adrenergic receptors and depressed postreceptor signaling pathways), alterations of intracellular calcium trafficking, blunted calcium sensitivity of contractile proteins, and mitochondrial dysfunction. Sepsis-induced myocardial dysfunction is not associated with significant cell death, and complete recovery occurs in survivors within two weeks [11].

**Figure 2:**
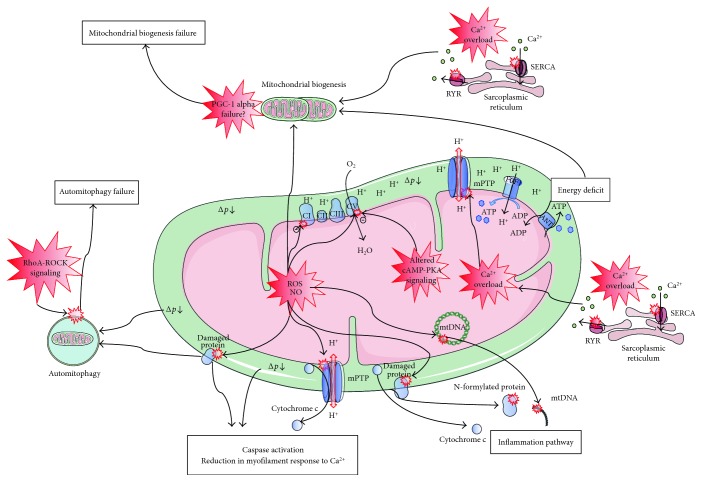
Mitochondrial disorders in septic cardiomyopathy. Increased reactive oxygen species (ROS) and nitric oxide (NO) production may cause direct oxidative or nitrosative damage and inhibition of oxidative phosphorylation (OXPHOS) complexes, leading to decreased O_2_ consumption and proton-motive force between the intermembrane space and the matrix (∆p). Reduced calcium (Ca^2+^) uptake by and increased Ca^2+^ leakage from the sarcoplasmic reticulum result in cytosolic and mitochondrial Ca^2+^ overload. Increased ROS/NO production, along with Ca^2+^ overload trigger the opening of the mitochondrial permeability transition pore (mPTP). This results in mitochondrial uncoupling of adenosine triphosphate (ATP) synthesis from O_2_ consumption (i.e., OXPHOS uncoupling) and a decreased ∆p. Altered mitochondrial cyclic adenosine monophosphate (cAMP) protein kinase A (PKA) signaling also promotes OXPHOS uncoupling and decreased ∆p. Decreased ∆p leads to ATP synthase (FOF1) inhibition and energy deficit. The mPTP opening and other mechanisms, as yet poorly described, induce externalization of mitochondrial components to the cytosol and the extracellular space that activates the inflammation pathway. Decreased ∆p, the presence of oxidized proteins, and externalization of mitochondrial components in the cytosol activate intrinsic apoptosis, leading to reduction in myofilament response to Ca^2+^. Although ∆p and the presence of oxidized proteins activate auto-mitophagy, RhoA-ROCK activation results in automitophagy failure. As increased ROS production, cytosolic Ca^2+^ overload and energy deficit activate mitochondrial biogenesis, and peroxisome proliferator-activated receptor *γ* coactivator 1 alpha (PGC-1 alpha) disorders result in mitochondrial biogenesis failure. Overall, increased inflammation, energy deficit, reduced myofilament response to Ca^2+^, impaired automitophagy, and failure in mitochondrial biogenesis are the features of mitochondrial disorders in septic cardiomyopathy. ADP: adenosine diphosphate; CI, CII, CIII, and CIV: the four complexes in the mitochondrial respiratory chain; mtDNA: mitochondrial DNA; RYR: ryanodine receptor; SERCA: sarcoendoplasmic reticulum Ca^2+^ pump.

Cardiomyocytes are characterized by a high density of mitochondria tightly packed between the sarcomeres and in the subsarcolemmal area [12]. Cardiac mitochondria are responsible for generating energy in the form of adenosine triphosphate (ATP) through oxidative phosphorylation (OXPHOS) and are crucial for cardiac function. Although mitochondrial impairment has been described in both human sepsis [13] and septic cardiomyopathy [14] for more than 15 years, mitochondria-targeted management is still absent from current clinical practice. A better understanding of mechanisms and any causative involvement of mitochondrial dysfunction in septic cardiomyopathy may guide future treatments in this field. Recent progress in sepsis research has increased our understanding of the underlying molecular alterations that contribute to cardiac dysfunction, including those related to mitochondrial dysfunction. In the following sections, we will review the evidence for the role of mitochondrial dysfunction in septic cardiomyopathy (Figure 2).

## 2. Ultrastructural Abnormalities of Myocardial Mitochondria in Sepsis

Mitochondria are intracellular organelles with a double membrane. This structure permits and is indeed essential for the mitochondrial production of the bulk of energy needed by the cell for normal function. Proton transfer from the matrix, across the inner membrane into the intermembrane space, is driven by the electron transfer chain and leads to phosphorylation of adenosine diphosphate (ADP) to ATP. This cascade of reactions requires mitochondria to be the most impermeable organelle in the cytoplasm and necessitates homeostasis of mitochondrial organization. When either inner or outer membrane permeability is disrupted, mitochondria change shape and this is associated with dysfunction and the release of content into the cytoplasm. Assessment of morphological characteristics such as swelling, disruption of cristae, and the length or density of mitochondria represents an easy way to identify mitochondrial dysfunction in various tissues.

**Figure 3:**
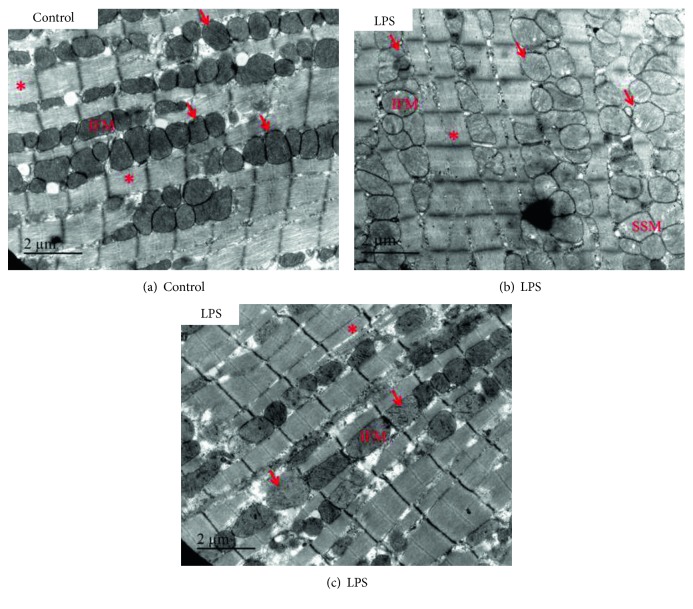
Altered cardiac mitochondrial morphology in lipopolysaccharide- (LPS-) treated mice. Representative, longitudinal electron microscopy micrographs of the left ventricle from control (a) and LPS-treated (b, c) mice. Six hours after LPS administration, cardiac mitochondria displayed abnormalities such as swelling, loss of cristae, and cleared matrix. Representative areas of mitochondrial clustering (arrow) and myofibrils (asterisk) are indicated. IFM: interfibrillar mitochondria are arranged along the myofibrils; SSM: subsarcolemmal mitochondria.

The first description of morphological impairments of cardiomyocyte mitochondria was made in an animal model of septic cardiomyopathy in 1994 [14]. Recently, in a rat model of endotoxin-induced peritonitis, Vanasco et al. [15] reported both a dysfunction of OXPHOS by determining respiration and electron transfer in isolated cardiac mitochondria and an alteration of cardiac mitochondrial ultrastructure by transmission electron microscopy in left ventricle myocardium. A half-maximal effect on respiration was observed about 4 h after LPS administration. Simultaneously, the percentage of damaged mitochondria increased in 6 h from 1.4% to 6.3%. It is worth recalling that impaired biochemical function seems to precede the structural damage. The effects on heart mitochondrial structure of LPS administration are illustrated in Figure 3. After 6 h to 18 h of LPS injection, cardiac mitochondria display abnormalities such as swelling, loss of cristae, cleared matrix, internal vesicles and rupture of the inner and outer membranes, and alterations that persisted up to 24 h [15].

Cardiac tissue remains difficult to study in humans, requiring invasive procedures, while skeletal muscle is less problematic in this regard. Using an established scoring system for mitochondrial structure damage, ranging from 0 (no abnormalities) to 4 (severe abnormalities), Fredriksson et al. studied a cohort of 10 patients with sepsis-induced multiple organ failure. In intercostal and leg muscle, they observed a trend of increased abnormalities in the septic group compared with controls. The same results were evident in both subsarcolemmal and intermyofibrillar mitochondria [16]. Only one study has evaluated sepsis-related changes in human cardiac mitochondrial ultrastructure [17], focusing on the mechanistic basis of cardiac and renal dysfunction in patients who died from septic shock. Electron microscopy on 17 septic hearts revealed a mitochondrial injury score of 3 in 6 patients (35%), whereas abnormalities were absent in the control group comprising cardiac donor hearts for transplant [17].

Ultrastructural abnormalities of mitochondria are associated with mitochondrial dysfunction and, through disruption of OXPHOS, a decreased capacity for ATP production—the primary role of mitochondria.

## 3. Oxidative Phosphorylation Disorders of Myocardial Mitochondria in Sepsis

Mitochondrial OXPHOS is responsible for over 90% of total oxygen consumption and ATP generation in the body. The mitochondrial respiratory chain is organized into four individual enzyme complexes in the inner membrane (complex I, complex II, complex III, and complex IV) that create an electrochemical gradient of protons between the matrix and the intermembrane space [18, 19]. Beta-oxidation of fatty acids and the Krebs cycle provide reduced nicotinamide adenine dinucleotide (NADH) and flavin adenine dinucleotide (FADH2) to the respiratory chain. NADH and FADH2 provide electrons to complex I and complex II, respectively. Electron transport from complex I and/or complex II to complex III then on to complex IV leads to proton translocation from the matrix to the intermembrane space and reduction of O_2_ to H_2_O. The oxidative activity of the respiratory chain thus leads to an accumulation of protons in the intermembrane space and creates a proton motive force (∆p) between the intermembrane space and the matrix [19, 20]. The ATP synthase (F0F1), located in the inner mitochondrial membrane, is driven to produce ATP by this ∆p and the passage of H^+^ from the intermembrane space to the matrix. Thus, F0F1 couples oxidative activity and O_2_ consumption of the respiratory chain to ATP regeneration. The ADP/ATP ratio is the physiologic activator of OXPHOS [21]; moreover, numerous posttranslational changes in respiratory complexes can subtly regulate OXPHOS and ATP production [22].

Alterations of organ function during sepsis are associated with OXPHOS dysfunction. Brealey et al. reported an association between shock severity, OXPHOS dysfunction (decreased activity of Complex I), ATP depletion, intracellular antioxidant depletion (reduced glutathione), and NO production in skeletal muscle tissue of patients with septic shock [13]. Mitochondrial dysfunction and reduced ∆p have also been described in septic patients and were associated with clinical disease severity in peripheral blood monocytes [23] and platelets [24]. Reduced activity and quantity of platelet complex IV were independent predictors of 6-month mortality in septic patients [25]. Reduced ADP-stimulated mitochondrial respiration (state 3) in peripheral blood mononuclear cells has been associated with increased prevalence of organ failure and mortality [26].

Mitochondrial dysfunction in septic cardiomyopathy is also characterized by decreased rates of state 3 respiration in mice and rats [27–29]. Moreover, increased rates of ADP-independent mitochondrial respiration (state 4) have been demonstrated in septic hearts from murine models [27, 28, 30], indicative of proton leakage across the inner mitochondrial membrane bypassing F0F1 (i.e., OXPHOS uncoupling). Thus, the respiratory control ratio (RCR—i.e., the state 3/state 4 ratio) is decreased in murine models of septic cardiomyopathy. This decrease in RCR reflects a reduced capacity of the respiratory chain to maintain a constant gradient of protons and is also associated with decreased ∆p [27, 28, 31].

The principal mechanisms involved in OXPHOS disorders seen in septic cardiomyopathy include overproduction of reactive oxygen and nitrogen species, calcium overload, and altered cAMP-PKA signaling. Increased production of superoxide anions and NO can cause direct oxidative or nitrosative damage and inhibition of OXPHOS complexes, leading to reduced O_2_ consumption and decreased ∆p. Firstly, reactive oxygen species (ROS) and nitrogen species are produced in substantial excess during sepsis [32], partly by mitochondria [33]. Mitochondrial complex I and complex IV can be inhibited by ROS, NO, and other nitrogen species. While it has been established that complex IV inhibition is always reversible by NO [34], prolonged exposure to NO results in a gradual and persistent inhibition of complex I [35]. Mitochondrial production of ROS and NO contributes to mitochondrial dysfunction during sepsis in various tissues, including the heart [15, 30]. Secondly, the antioxidant systems seem to be inhibited: inhibition of mitochondrial complex I by NO was facilitated, *in vitro*, by a depletion of reduced glutathione [36]. LPS-related depletion of reduced glutathione occurred within 6 h in the cardiac mitochondria of rats [37]. The activities of manganese superoxide dismutase and glutathione peroxidase enzymes in cardiac mitochondrial fractions from septic rats decreased significantly within 4 h after bacterial challenge [38]. Thirdly, when superoxide anions and NO are produced simultaneously, they rapidly react together to yield the peroxynitrite anion, which may result in significant inactivation of F0F1-ATP synthase [39]. Furthermore, complex I inhibition appears to result from *S*-nitrosylation of critical thiols in the enzymatic complex [35]. The increased activity of the inducible mitochondrial NO synthase is responsible for increased mitochondrial peroxynitrite levels [40]. In a mouse model of cecal ligation and puncture, Escames et al. showed that increased oxidative stress and mitochondrial dysfunction were restored by genetic deletion of inducible NO synthase (which includes the mitochondrial isoform), as well as by melatonin treatment, a known inhibitor of inducible NO synthase [41]. Together, these results suggest a significant role for peroxynitrite in myocardial mitochondrial dysfunction in sepsis. Finally, ROS species can lead to a diverse array of both reversible and irreversible toxic modifications of proteins, lipids, or nucleic acids. This oxidative stress alters respiratory capacity by decreasing proteomic expression of several mitochondrial-encoded subunits of complex I, complex IV, and F0F1 [37]. In addition, activation of mitochondrial calpain-1 can disrupt F0F1, leading to increased mitochondrial ROS generation, further alteration of mitochondrial components and promotion of a proinflammatory response during endotoxemia [42].

Under pathological conditions, some protons return into the matrix while bypassing F0F1 via uncoupling proteins (UCPs) located in the inner mitochondrial membrane [43]. This results in OXPHOS uncoupling—the dissociation of ATP synthesis from O_2_ consumption in the mitochondria. The role of UCPs in sepsis remains controversial. In a rat model of cecal ligation and puncture, Roshon and colleagues found an increase in UCP_2_ mRNA levels concurrent with decreased cardiac work and mechanical efficiency [44], but no UCP_2_ protein was detected in the hearts 12 h after insult. Significant increases in levels of UCP_2_ and UCP_3_ mRNA were observed in the hearts of LPS-treated rats [37], while both mRNA and protein levels of UCP_2_ were increased in the myocardium in a canine model of endotoxin-induced shock [45]. In this model, Wang et al. found an association between elevated UCP_2_ expression and decreased ATP generation [45]. Conversely, increased UCP_2_ expression in cardiac mitochondria may produce only mild uncoupling that prevents ROS production without altering ATP generation. Indeed, Zheng et al. reported that H9C2 cells cultured with LPS and peptidoglycan showed increased mRNA expression of UCP_2_ and that this was associated with mitochondrial dysfunction [46]. These effects were worsened by silencing UCP_2_, suggesting that UCP_2_ may play a protective role in cardiomyocytes under septic conditions.

Permeability of the inner mitochondrial membrane is mediated by a voltage- and calcium dependent, cyclosporine A-sensitive, high-conductance channel called the mitochondrial permeability transition pore (mPTP). Mitochondrial calcium overload is the primary trigger of mPTP opening, but its sensitivity to this challenge is dependent on prevailing conditions [47]. In a rat model, Hassoun et al. reported that an increased mitochondrial calcium content (induced by LPS) was associated with reduced calcium uptake by and increased calcium leakage from the sarcoplasmic reticulum and it was also associated with decreased ∆p, mitochondrial uncoupling, altered state 3 respiration, and impaired RCR. Prevention of calcium leakage from sarcoplasmic reticulum by dantrolene prevented mitochondrial and cardiac contractile dysfunction, suggesting that mitochondrial calcium overload could contribute to myocardial mitochondrial dysfunction in sepsis [28]. In a mouse model of acute peritonitis, Larche et al. observed that improvement of mitochondrial function via inhibition of mPTP opening (with cyclosporine A or overexpression of the antiapoptotic protein Bcl-2) prevents mortality and heart dysfunction [27]. Inhibition of mPTP opening also prevented a sepsis-related decrease in ∆p and RCR in the septic heart. Overall, mitochondrial calcium overload and mPTP opening seem to be the main mechanisms of mitochondria uncoupling in septic cardiomyopathy.

Posttranslational changes in respiratory complexes may also be involved in sepsis-induced mitochondrial and contractile dysfunction in the heart. Cyclic adenosine monophosphate (cAMP) is produced within mitochondria by HCO_3_/CO_2_-responsive adenylyl cyclase, which couples CO_2_ generation in the Krebs cycle with OXPHOS activity [48–50]. Increased mitochondrial cAMP induces mitochondrial protein kinase A (PKA) activation. Thus, allosteric ATP inhibition of mitochondrial complex IV is prevented by reversible PKA-mediated phosphorylation of serine 58 of complex IV subunit IV-1. The mitochondrial cAMP-PKA pathway couples the Krebs cycle and OXPHOS activity to generate ATP. In a mouse model of acute peritonitis, Neviere et al. observed that sepsis-related cardiomyopathy was associated with impaired cAMP-PKA signaling, decreased complex IV phosphorylation of serine 58, decreased mitochondrial RCR, and left ventricle contractile dysfunction [51]. Ex vivo inhibition of phosphodiesterase 2A by mitochondria-permeant Bay 607550 prevented mitochondrial cAMP depletion, complex IV phosphorylation, OXPHOS uncoupling, and contractile dysfunction. *In vivo* administration of Bay 607550 did not prevent contractile dysfunction but restored energetic efficiency of the left ventricle in septic mice.

While the mitochondrial capacity for generating ATP is decreased in sepsis, mitochondrial dysfunction is not associated with significant myocardial necrosis in human septic shock [11]. Nonetheless, respiratory chain dysfunction is associated with contractile dysfunction and prevention of OXPHOS disorders prevents cardiac dysfunction in murine models of sepsis [27–31]. The importance of mitochondria in cellular homeostasis could provide a mechanistic explanation for the link between mitochondrial dysfunction and heart failure in septic cardiomyopathy [52].

## 4. Alteration of Mitochondrial Signaling in Septic Cardiomyopathy

Mitochondrial ROS production [53, 54], or externalization of other mitochondrial components in the cytosol (e.g., cytochrome c, SMAC/diablo, PGAM5, and mitochondrial DNA (mtDNA)) [55–57], may be considered to be a part of cellular signaling. These components are involved in both the apoptotic pathway and inflammation, key features in the pathophysiology of septic cardiomyopathy.

Mitochondria are critical players in the regulation of programmed cell death [58]. They modulate apoptosis during the initiation and regulation of the intrinsic apoptotic pathway, also known as mitochondria-mediated apoptosis. Caspase 9-dependent activation of executioner caspases such as caspase 3, 6, and 7 is a characteristic of the intrinsic pathway. Oxidative stress, OXPHOS dysfunction, and mPTP opening are the main triggers for mitochondria-mediated apoptosis. Initially, ROS-induced protein alterations lead to the accumulation of Nix and Bnip3 proteins on the outer mitochondrial membrane. Thus, Nix and Bnip3 recruit proapoptotic proteins from the cytosol (such as Bax and Bak) on the surface of mitochondria. Thereafter, Bax and Bak permeabilize the outer membrane to molecules such as cytochrome c which triggers the apoptotic pathway [57, 58]. Secondly, OXPHOS dysfunction and a decrease in the ∆p can lead to the cleavage of a mitochondrial phosphatase, PGAM5. Cleaved PGAM5 translocates to the cytosol where it activates the apoptotic pathway [56, 57]. Thirdly, the mPTP opening directly permeabilizes mitochondria and can release cytochrome c to the cytosol, activating the intrinsic apoptotic pathway. Finally, the extrinsic apoptotic pathway may also interfere with mitochondria-induced apoptosis. The extrinsic pathway can activate Bax and Bak, which directly permeabilize the outer mitochondrial membrane and trigger the intrinsic apoptotic pathway [59].

Nevière et al. first demonstrated the importance of apoptosis regulation in an experimental cardiomyopathy model of endotoxin-treated rat. Caspase 9 and 3 activities and the apoptosis pattern (DNA fragmentation or cytochrome c release) were enhanced in this experimental model. Cotreatment with a nonspecific caspase inhibitor not only reduced caspase activity and nuclear apoptosis but was also associated with a complete correction of endotoxin-induced myocardial dysfunction [60]. The same authors also demonstrated that specific inhibition of caspases 9 and 3 prevented reduction in myofilament responses to calcium, troponin T cleavage, and sarcomere destruction in endotoxin or septic serum-treated rats [61]. Mitochondrial activation of intrinsic apoptosis was also implicated in rats with post cecal ligation puncture-induced septic cardiomyopathy. In this model, prevention of cardiomyocyte dysfunction was also prevented by inhibition of the intrinsic apoptotic pathway using cyclosporine A or Bcl-2 overexpression [27].

All these experimental data converge to implicate mitochondrial upregulation of the intrinsic apoptotic pathway in the genesis of septic cardiomyopathy. However, while the mitochondrial apoptotic pathway has not been evaluated in human sepsis, the importance of its activation seems overestimated by experimental models as apoptosis-related death of cardiomyocytes does not significantly occur in human sepsis [11, 17]. Whether caspase activation and/or caspase-induced cleavage of troponin T occurs in the cardiomyocytes of patients with septic shock remains unknown.

Lovett et al. [62] suggested that myocardial cell depression may be due to circulating myocardial depressant factors. Parrillo et al. later confirmed the existence of depressant factors in human sepsis, showing that serum obtained from septic shock patients and incubated *in vitro* with cardiomyocytes decreased the extent and velocity of myocyte shortening. Removal of the preparation by washing with serum obtained from nonseptic patients rapidly restored the contractile force of cardiomyocytes [63]. Proinflammatory cytokines (TNF-*α* and IL1-*β*) and NO were the first myocardial depressant factors described [64]. Damage-associated molecular patterns (DAMPs) are endogenous molecules released in cytoplasm or the circulatory torrent in response to cellular stress, whereupon they can react to pattern recognition receptors also called host defense receptors of innate immunity. From the “Danger model” developed by Matzinger, DAMPs can trigger the immune system and inflammation pathway [65, 66], engaging a defensive reaction to cellular damage. A potential role has been proposed for DAMPs in sepsis-induced organ dysfunction such as that seen in septic cardiomyopathy. For instance, HMGB1, a nuclear DAMP, can induce cardiac dysfunction via TLR4 interaction and consequently enhance oxidative stress and impairment of cardiac excitation-contraction coupling [9, 67]. Mitochondria can also react to infectious or inflammatory aggression by liberating numerous components, collectively known as mitochondrial DAMPs (mtDAMPs), into cytoplasm and the circulatory torrent. Once there, mtDAMPs can interact with pattern recognition receptors and induce a proinflammatory response to damage, thereby implicating mitochondria in the regulation of danger signaling [68]. The structural homology of mtDNA (containing CpG-nonmethylated DNA and formylated proteins) underpins the proposed bacterial origin of mitochondria (the endosymbiotic theory) and partially explains its recognition by pattern recognition receptors. The mtDNA is the most studied mtDAMPS in experimental or human sepsis, but other mtDAMPs have been described such as cytochrome c or N-formyl proteins [69, 70]. Although conflicting data exist, owing largely to technical issues around the detection of free plasmatic mtDNA, most experimental and human results report elevated free plasmatic circulating mtDNA in sepsis [71–73]. In addition, quantitative expression seems to correlate with severity and mortality in sepsis in a way similar to a large number of diseases encountered in critical care [74, 75]. However, the mechanism of mtDNA externalization in septic subjects remains unclear. Mechanism does not seem as simple as in trauma patients and may implicate necrosis, necroptosis, apoptosis, or autophagy process [76–78]. Mitoptosis, a recently described caspase-independent mechanism by which mitochondria undergo extensive fragmentation, is another potent mechanism for the release of mitochondrial content to the cytoplasm or plasma [79].

It is nowadays clear that circulating mtDNA can contribute to systemic inflammation [80], neutrophil activation [81], or even postseptic organ dysfunction of the immune system [82], lung [83–85], or kidney [86]. No data are yet available on the putative induction role of mtDNA in septic cardiomyopathy, but Oka et al. described mtDNA as a candidate inducer of cardiac dysfunction in an experimental mouse model of heart failure [87]. Likewise, cytochrome c and N-formyl protein have been shown capable of injuring cardiomyocytes and provoking cardiac dysfunction in various models [69, 70]. While mtDAMPs may be a myocardial depressant factor, new experimental data are necessary to better understand their role in septic cardiomyopathy.

## 5. Mitochondrial Biogenesis and Mitophagy in Septic Cardiomyopathy

The quality and quantity of mitochondria largely depend upon both mitochondrial biogenesis and the autophagy/mitophagy system. Mitochondrial biogenesis is mainly activated in cases of low ATP production, oxidative stress, and calcium overload. In the presence of biogenesis activators, peroxisome proliferator-activated receptor *γ* coactivator (PGC-1 alpha) and nuclear transcription factors such as nuclear respiratory factors (NRFs), estrogen-related receptors (ERRs), and peroxisome proliferator-activated receptors (PPARs) are overexpressed and activated [88]. The transcription of nuclear genes coding for metabolic enzymes, respiratory chain proteins, mitochondrial transcription factors (mTFs), and other mitochondrial proteins is thus activated. In humans, over 97% of mitochondrial proteins are translated from nuclear mRNA and subsequently translocated to mitochondria. The mTFs A, B1, and B2 activate the replication of mtDNA and transcription of the 37 mitochondrial genes, essential for mitochondrial activity, and permit adequate interplay between nuclear and mitochondrial genomes during mitochondrial biogenesis. While insufficient biogenesis limits ATP production and induces cell necrosis [89], an excessive production of mitochondria leads to disruption of myofibrils and cardiomyocyte dysfunction [90]. Mitochondria undergo continuous oxidative stress due to oxidative activity of the mitochondrial respiratory chain. Production of superoxide anion, hydrogen peroxide, and hydroxyl radical leads to impairment of protein structure, peroxidation of membrane lipid, and alteration of mtDNA [19]. Nonspecific autophagy and mitochondria-specific autophagy (i.e., mitophagy) remove damaged and dysfunctional mitochondria [91]. Mitophagy mainly involves activation of PINK1/Parkin, PGAM5/FUNDC1, or the Nix/Bnip3 pathways. A decreased ∆p leads to accumulation of PINK1/Parkin and PGAM5/FUNDC1 on the outer mitochondrial membrane. The oxidation of mitochondrial proteins drives to Nix/Bnip3 accumulation on the cytosolic surface of mitochondria. Thus, PINK/Parkin, PGAM5/FUNDC1, and Nix/Bnip3 activate mitophagy and removal of dysfunctional mitochondria [88]. Autophagy and mitophagy can limit accumulation of depolarized mitochondria, generation of superoxide anion, hydrogen peroxide, and hydroxyl radical, and the release of mitochondrial content into cytosol or the extracellular space. There exists significant interplay between mitophagy and mitochondrial biogenesis. On the one hand, biogenesis activation by mitophagy permits renewal of the degraded organelles [92]; on the other hand, mitophagy activation by mitochondrial biogenesis creates space for new functional organelles [93].

In the septic heart, there is an early (H6 to H24) decrease in mitochondrial mass with in parallel, early activation of biogenesis pathways [15, 37, 94, 95]. In rat cardiomyocytes, endotoxins induced significant increases in biogenesis markers such as PGC-1 alpha, NRF-1, and mTFs [96]. Autophagy was also stimulated in this model, as indicated by an increased expression of microtubule-associated light chain 3 (LC3). In humans, survival was associated with activation of mitochondrial biogenesis (assessed by PGC-1 alpha, NRF-1, and mTFA) in the skeletal muscle of septic patients [97]. Importantly, manganese superoxide dismutase expression (representing antioxidant system activity) was associated with an increase in these markers of biogenesis. Unsurprisingly, patients who died from sepsis had significantly decreased expression of nuclear genes coding for Krebs cycle enzymes and respiratory chain proteins [98]. The exact mechanisms by which transcription of nuclear genes coding for mitochondrial proteins is altered in patients dying from sepsis remain unclear. Nevertheless, ROS-induced damage of mitochondrial DNA might be responsible for a relative insufficiency of mitochondrial transcription and mitochondrial biogenesis in septic subjects [37].

Carbon monoxide can cause cardiac ischemia with an inhibition of the respiratory chain together with an increase in cardiac contractility [99]. Interestingly, as exposure to elevated doses of carbon monoxide increased mortality in a mouse model of sepsis, while exposure to low doses activated mitochondrial biogenesis in the heart and prevented ∆p alterations in cardiac mitochondria, as well as acute mortality [31]. Thus, minor inhibition of the respiratory chain may, to some extent, play a beneficial role in septic models [100, 101].

Nevertheless, partial restoration of cardiac mitochondrial mass is not always accompanied by improvement of mitochondrial function in acute endotoxemia [15]. Overexpression of PGC-1 alpha in the heart could be deleterious. Transgenic mice overexpressing PGC-1 alpha displayed a reversible cardiomyopathy: this cardiomyopathy was characterized by an increase in ventricular mass (eccentric hypertrophy) and chamber dilation (echocardiographic data) [102]. In the neonatal stages of this model, overexpression of PGC-1 alpha increased the number and size of mitochondria; in adult mice, this overexpression only modestly increased the number of mitochondria, leading to changes in mitochondrial ultrastructure (e.g., apparition of vacuoles and granular inclusions) and cardiomyopathy.

A relative insufficiency of autophagy/mitophagy may impede clearance of dysfunctional mitochondria despite appropriate biogenesis in the septic heart. In a LPS injection model, autophagy was activated in the mouse heart [29]. In this model, inhibition of Drp1 phosphorylation by fasudil (a ROCK inhibitor) further activated autophagy and prevented cardiac and mitochondrial dysfunction. Similarly, autophagy stimulation in a model of cecal ligation and puncture could restore both cardiac function and cardiac ATP production [103], whereas inhibition of autophagy in a murine peritonitis model increased apoptosis and hepatic injury [104]. Carbon monoxide exposure may regulate both mitochondrial biogenesis and autophagy. Indeed, low doses of carbon monoxide prevented acute mortality in a murine peritonitis model via the systemic enhancement of autophagy and phagocytosis [105]. Mice deficient for the autophagic protein Becn1 (Becn1+/−) were more likely to die from sepsis and be unresponsive to carbon monoxide therapy. The role of cardiac mitophagy per se in sepsis has been evaluated in only one animal study [106]. Sublethal doses of endotoxin led to early mitophagy activation, transitory cardiac dysfunction (assessed by isolated heart preparation), and reversible alteration of mitochondrial respiration in wild-type mice. Conversely, Parkin−/− mice displayed only partial recovery of mitochondrial and cardiac function, despite residual mitophagy activation. Therefore, early and complete activation of mitophagy pathways seem essential for recovery of mitochondria and cardiac function during septic cardiomyopathy.

Overall, the relative and/or absolute insufficiency of mitochondrial biogenesis and auto-/mitophagy may be important for contractile and mitochondrial dysfunction in the septic heart. Nonetheless, the precise mechanisms of mitochondrial biogenesis alteration and mitophagy insufficiency are still poorly understood and require further investigation.

## 6. Mitochondrial Genetic and Sepsis

According to the endosymbiotic theory, mitochondria originated from aerobic free-living bacteria-like organisms (alpha-proteobacteria). At some point in evolution, they were engulfed by primitive, nucleated anaerobic cells to form symbiotic, eukaryotic cells [107]. The organizational features supporting endosymbiotic theory are the presence of a double membrane and its own mtDNA. The mtDNA is a small, circular, double-stranded molecule only 16.5 kb in length. The 37 genes of the human mitochondrial genome encode 13 essential components of the OXPHOS system (i.e., complex I, complex III, complex IV, and FOF1), 22 transfer RNA, and 2 ribosomal RNA [108]. Nevertheless, nuclear genes code for the majority of mitochondrial proteins subsequently translocated into mitochondrion from cytosolic ribosomes. The mtDNA is almost exclusively inherited from the maternal line. A high random mutation rate of mtDNA can be due to the lack of protective histones, inefficient DNA repair mechanisms, and mutagenic effects of mitochondria-generated ROS [109]. Consequently, a large number of single-nucleotide polymorphisms of mtDNA have accumulated among maternal lineages and have diverged as human populations dispersed more widely to different geographical regions of the world. These specific single-nucleotide polymorphisms are known as mtDNA haplogroups [110]. Nine haplogroups have been successively described (H, J, T, U, K, V, W, I, and X), the majority of the European population belonging to haplogroup H (44%) [111].

A longitudinal clinical and genetic study of 150 patients with septic shock revealed that haplogroup H patients presented proportionally better survival than other haplogroups at 28 days, upon hospital discharge and at a six-month follow-up [112]. Furthermore, the Spanish sepsis group of researchers reported a protective effect of haplogroup H on sepsis incidence in a study of 240 patients with postoperative sepsis [113]. Haplogroup H is the most recent addition to the group of European mtDNA but, perhaps paradoxically, is the most common: indeed, increased survival after septic shock may provide one explanation for this. The hypothesis of a direct, functional consequence of improved mitochondrial efficiency arising from this clinical report was subsequently tested by Amo et al. [114]. Using a transmitochondrial cytoplasmic hybrid cell, they found no difference in mitochondrial bioenergetic capacities or coupling efficiencies when comparing mitochondria from haplogroup H with those from haplogroup T. Haplogroup H survival protection remains poorly understood, and haplogroup effects on mitochondrial proliferation or signaling processes have not yet been fully explored. Other research groups have described the potential consequences of belonging to other haplogroups: for example, haplogroup JT was associated with increased survival in a prospective cohort of 96 patients with severe sepsis and an increased complex IV activity in patients from this haplogroup relative to others [115, 116].

Together, these data indicate a potential effect of genetic haplogroup variants on survival in sepsis cases. The association with functional mitochondrial activity remains unclear; however, no data yet exist on the consequences for cardiac mitochondria during sepsis.

## 7. Conclusions

Cardiac dysfunction is common in patients suffering from sepsis and septic shock. Mitochondrial dysfunction takes part in the pathophysiology of septic cardiomyopathy and is associated with patient outcome. Respiratory chain disorders, the role of dysfunctional mitochondria in cellular homeostasis, and insufficient renewal of mitochondria are deleterious mechanisms in both development and persistence of cardiac dysfunction in septic subjects. The weight of evidence for the involvement of mitochondrial dysfunction in septic cardiomyopathy makes it a potential target for future treatment of sepsis. Nevertheless, the precise mechanisms and any causative role for mitochondrial impairments in human cardiomyopathy are still poorly understood and require further investigation before clinical application.
